# Microstructural Stability of 316 L Produced by Additive Manufacturing for Nuclear Applications

**DOI:** 10.3390/ma19081610

**Published:** 2026-04-17

**Authors:** Roberto Montanari, Alessandra Palombi, Maria Richetta, Giulia Stornelli, Alessandra Varone, Ali Zahid

**Affiliations:** 1 Department of Industrial Engineering, University of Rome Tor Vergata, 00133 Rome, Italy; roberto.montanari@uniroma2.it (R.M.); alessandra.palombi@uniroma2.it (A.P.); richetta@uniroma2.it (M.R.); alessandra.varone@uniroma2.it (A.V.); 2 Department of Engineering, University of Perugia, 06123 Perugia, Italy; giulia.stornelli@unipg.it

**Keywords:** austenitic stainless steels, 316 L, laser powder bed fusion, microstructure

## Abstract

Additive manufacturing (AM) represents a quite interesting technology for manufacturing components of nuclear reactors. This work investigated the microstructural stability of 316 L steel fabricated via Laser Powder Bed Fusion (L-PBF) from room temperature to 650 °C. Despite the reduced susceptibility of the material to sensitization owing to its low carbon content, temperature variations may induce deleterious effects in nuclear safety-critical components. In as-printed condition, the microstructure is not stable and undergoes significant changes induced by thermal cycling up to 650 °C in Mechanical Spectroscopy (MS) tests: the typical melt-pool pattern disappears, a population of equiaxed grains substitutes the original ones elongated in the build direction, the average size of the cells forming a finer sub-structure inside the grains increases, texture changes, and the excess of vacancies induced by the rapid cooling is recovered. Although the current literature reports that the microstructure is stable up to 500 °C, MS results indicate that the aforesaid irreversible phenomena start at a lower temperature (~230 °C). The present results suggest that the microstructure of the printed material must be stabilized through suitable heat treatments before its application in structural components for nuclear reactors.

## 1. Introduction

In recent years, extensive research has been ongoing to investigate the feasibility of additive manufacturing (AM) for components of nuclear reactors. AM is revolutionizing the way metallic alloys are produced and has opened new perspectives in various industrial sectors, such as the ability to manufacture complex shapes and structural light-weighting, thanks to the implementation of topological optimization [1] and cost reduction [1,2,3]. Among the materials of nuclear interest, 316 L steel is one of the most studied due to its large-scale use in different structural components [4,5,6] and in heat exchangers [7] in nuclear plants, which can be exposed to temperatures in the range 300–600 °C. Specifically, in the framework of the EU DEMO reactor design, AISI 316 L has been selected for the fabrication of cooling tubes and manifolds for the Vertical Targets (VTs), and for the Vacuum Vessel structures, where it must maintain structural integrity under significant nuclear heat and radiation damage [8].

A comprehensive review of AM techniques and challenges, and the future research directions for producing 316 L components has been provided by D’Andrea [9].

Printed 316 L exhibits a hierarchical austenitic microstructure consisting of grains of micrometric size containing a finer cellular structure. The high cooling rates (10^5^–10^8^ K/s) [10,11,12] and high thermal gradients [13] involved in the AM process give rise to residual stresses, micro-inclusions, and complex, heterogeneous microstructures that are out-of-thermodynamic equilibrium, much finer and quite different from those obtained from traditional techniques, such as casting and forging [14,15,16]. These microstructural features affect mechanical properties and change with solidification rate, depending on process parameters (laser power, scanning speed, hatch distance, layer thickness, and scanning strategy) [17,18,19,20]. In fact, smaller cell size consistently enhances the mechanical properties, especially yield stress and ultimate tensile strength. Moreover, the heterogeneous dislocation distribution in as-printed material produces intragranular residual stresses with pronounced tension–compression asymmetries in yield strength and work hardening [21].

The enhancement of mechanical behavior is generally attributed to the solute segregation and cellular sub-structure [22,23,24,25,26,27,28]; however, in spite of the great efforts devoted to studying this issue, some aspects about cell formation and deformation mechanisms are still debated (e.g., see refs. [29,30,31]). Dépinoy [32] investigated 316 L steel processed by Laser Powder Bed Fusion (L-PBF) and found that the dislocation patterns, which strongly depend on process parameters, are closely related to the periodicity of micro-segregation. Interdendritic dislocations are not geometrically necessary; namely, they do not stem from the solidification process but arise from thermo-mechanical stresses during cooling of the solid. Moreover, different strengthening mechanisms are active depending on the dislocations structures. These results are supported by the work of Barkia et al. [33], who demonstrated that the deformation mechanisms are strongly affected by the initial dual-scale cellular structure, consisting of large dislocation cells with chemically segregated cell walls, encapsulating smaller zones, which are chemically homogeneous. At low and medium strain levels, the primary deformation mode is dislocation slip across the dislocation cells’ interiors and the formation of micro-bands, while plastic deformation at higher strain is primarily controlled by mechanical nano-twins.

It is generally acknowledged that, in comparison with standard 316 L, the steel produced by L-PBF exhibits superior performance at elevated temperatures [33,34]; however, the non-equilibrium microstructure might evolve during service life in nuclear reactors. Thus, recently, particular attention has been paid to investigating its thermal stability. Chen et al. [35] found that nano-sized silicates precipitate after annealing at 400 °C, with a 10% increase in yield strength. In contrast, annealing at higher temperatures results in a decrease in strength, attributed primarily to the thermal instability of the cell structure in the as-manufactured material. It is reported in the literature that the cellular structure and precipitates (σ phase, M_23_C_6_ carbides, and Mo_3_Si silicides) remain stable up to 600 °C for a long annealing time of up to 400 h, while local cell coarsening was observed after 100 h at 700 °C [19,20]. Following heat treatments at temperatures higher than or equal to 1100 °C, partial phase transformation of austenite into ferrite, growth of subgrains inside the micrometric grains, and nucleation of the σ phase were detected [36,37]. Recrystallization also occurs at 1100 °C and above, with kinetics depending on the original microstructure: finer grains, lower density of low-angle grain boundaries (LAGBs) and higher precipitate fraction slow down the process [38]. Low-Cycle Fatigue tests conducted at 550 °C highlighted a softening behavior of 316 L produced by L-PBF that is related to three different phenomena: coarsening of the cellular sub-structure, crystallographic texture evolution, and a decrease in geometrically necessary dislocations’ (GND) density and LAGBs during fatigue tests [39,40].

Within the framework of a broader research campaign, carried out by the EUROfusion Consortium and addressing different topics (process parameter optimization, mechanical properties, etc.), the present work focuses on the microstructural stability of 316 L steel produced by L-PBF, the most widely adopted AM technology, with particular attention being given to the temperature range where possible microstructural changes occur. Besides being analyzed through more conventional techniques, the samples were subjected to Mechanical Spectroscopy (MS) tests at temperatures ranging from room temperature to 650 °C in order to assess the stability of the as-built microstructure. MS is a dynamic technique providing specific information about the material characteristics that cannot be obtained otherwise; it evidences anelastic phenomena, which are quite sensitive in terms of revealing small microstructural variations. The theory of anelasticity is illustrated in the book by Nowick and Berry [41], while a collection of the literature data is reported in ref. [42]. MS has been already successfully used by some of the present authors to investigate the AlSi10Mg alloy produced by L-PBF and obtain information about small variation oin its microstructural features [43].

## 2. Material and Methods

Samples of 316 L steel were fabricated using a commercial powder supplied by GE Additive (West Chester, OH, USA), whose nominal chemical composition (as provided by the manufacturer) is reported in Table 1. The samples were produced by L-PBF on a Concept Laser M2 Cusing (GE Additive, West Chester, OH, USA) system equipped with a single-mode CW ytterbium-doped fiber laser (emission wavelength 1070 nm) and a metallic build platform.

**Figure 1:**
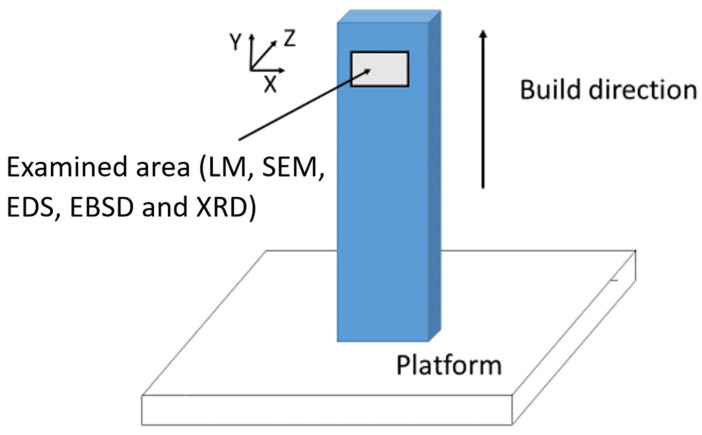
Schematic view of the printing set-up of a single sample. The area subjected to microstructural examinations is indicated.

The raw metal powder used to print the samples consisted of spherical particles ranging from d_10_ = 18.17 μm to d_90_ = 45.44 μm in diameter and the process was performed under inert Ar atmosphere (residual O < 0.2%). Figure 1 shows a schematic view of the printing set-up of a single sample.

The process parameters were as follows: laser power 180 W, scanning speed 600 mm/s, laser spot diameter 120 μm, hatch distance 105 μm, and layer thickness 25 μm. Each layer was divided into 5 × 5 mm^2^ squares that were scanned according to the same bi-directional, alternated and rotated pattern described in [44,45]. The printed reeds (40 mm × 7 mm × 0.7 mm) were then cut from the building platform and subjected to mechanical polishing to reduce the surface roughness to a final value of *R* = 1 µm. The steel was not subjected to heat treatments after printing.

The printed samples were examined through Light Microscopy (LM), Scanning Electron Microscopy (SEM), Energy Dispersive Spectroscopy (EDS), Electron Backscattered Diffraction (EBSD) and X-Ray Diffraction (XRD), and Mechanical Spectroscopy (MS) tests.

MS experiments were performed on as-built samples to measure the damping parameter (*Q*^−1^) and dynamic modulus (*E*) from room temperature to 650 °C. To ensure high signal resolution in MS measurements, a heating/cooling rate of 1.5 °C/min was adopted. The tests were carried out through an automated vibrating reed analyzer (VRA 1604, CANTIL s.r.l., Bologna, Italy) [46] operating in resonance conditions. The samples were mounted in cantilever geometry and put into resonance by an electrostatic excitation. The frequency was in kHz range, the pressure in the measurement chamber < 10^−6^ mbar, and the strain amplitude 10^−5^. The dynamic modulus was determined from the resonance frequency *f* through Equation (1):

(1) $$E=\frac{48\pi^{2}\rho L^{2}}{m^{4}h^{2}}f^{2}$$

where *m* = 1.875 is a constant, *L* and *h* the length and thickness of the sample, and *ρ* the material density. The relative standard deviation in the modulus measure depends on the ratio between surface roughness and sample thickness [47], and it is ±0.003 under the conditions of present experiments.

The damping parameter *Q*^−1^ was determined by the logarithmic decay of the flexural vibrations (Equation (2)) when the electrostatic excitation was switched off:

(2) $$Q^{-1}=\frac{1}{p\pi }\ln(\frac{A_{n}}{A_{n+p}})$$

being *A_n_* and *A_n_*_+*p*_ the amplitudes of the *n*-th and *n* + *p*-th oscillation.

The microstructure after MS test was investigated through LM, SEM, EDS, EBSD and XRD; the examined area is shown in Figure 1.

The samples used for LM (Union Optical Co., Ltd., Tokyo, Japan) and SEM (ZEISS Leo 1530, Jena, Germany) observations were prepared through metallographic polishing by means of grinding papers with SiC abrasive particles of decreasing size (up to n. 4000), plus a final step employing a cloth with a suspension of diamond particles (3 µm). Finally, they were etched in a solution of 15 mL HCl, 10 mL HNO_3_, 10 mL CH_3_COOH and 2 drops of glycerol for 90 s.

EBSD measurements were performed by means of a FEG-SEM (Ultra-Plus Carl-Zeiss, Oberkochen, Germany) equipped with an EBSD detector (C Nano Oxford Instruments, High Wycombe, Buckinghamshire, UK), using a 0.5 μm scanning step size. The technique was employed in order to evaluate the fractions of high-angle grain boundaries (HAGBs), low-angle grain boundaries (LAGBs), as well as the kernel average misorientation (KAM). Moreover, image analysis was performed on EBSD maps to determine the average grain size by using dedicated software (AlexaSoft X-Plus, Florence, Italy).

XRD patterns were collected from the material in the as-built condition and after MS test using the Mo-Kα radiation (λ = 0.07093 nm) in the 2Θ angular range 15–50° with 2Θ steps of 0.05° and counting time of 5 s per step. High precision peak profiles of the most intense reflections were recorded with 2Θ steps of 0.005° and counting time of 20 s per step. The relative intensities of the peaks in each pattern were compared with those of Fe-γ reported in the database JCPDS–file 6-696 [48] and corresponding to a material with randomly oriented grains. The comparison allowed us to evaluate the presence of preferred grain orientations.

## 3. Results

**Figure 2:**
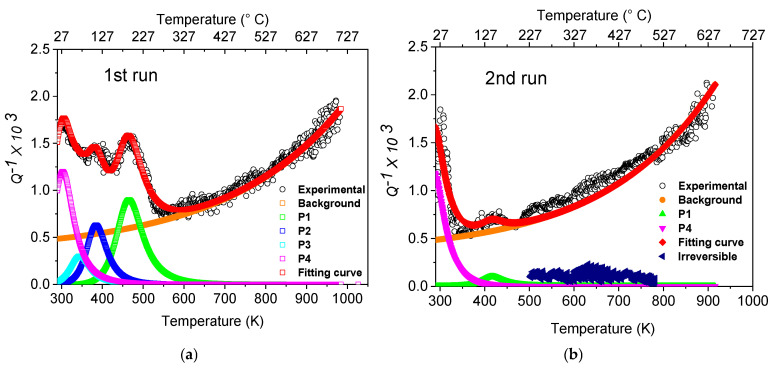
*Q*^−1^ vs. temperature curves obtained from the as-printed steel in successive MS tests: 1st (**a**) and 2nd (**b**) runs. According to Equation (3), experimental data have been fitted (red curve) as the sum of an exponential background and four Debye peaks (*P*1, *P*2, *P*3 and *P*4).

Figure 2a,b show the *Q*^−1^ curves obtained from the as-printed steel in two successive MS test runs.

The *Q*^−1^ curve in the 1st run has been fitted as the sum of an exponential background (*Q*^−1^*_back_*) and four Debye peaks (Equation (3)):

(3) $$Q^{-1}=\sum_{n=1}^{4}\frac{\Delta_{n}}{2}\sec\;h\left[\left(\frac{H_{n}}{R}\right)\left(\frac{1}{T}-\frac{1}{T_{n}}\right)\right]+Q_{BACK}^{-1}$$

where *R* is the gas constant, the temperature *T_n_* the central position, *H_n_* the activation energy and *Δ_n_* = 2*Q*^−1^*_max_* the relaxation strength of the n-th peak. Relaxation peaks occur when the following condition, expressed by Equation (4), is satisfied:

(4) $$\omega \tau =\omega \tau_{0}e^{H/RT}=1$$

where ω = 2πf, τ is the relaxation time and τ_0_ its pre-exponential factor. Therefore, the activation energies of *Q*^−1^ peaks and pre-exponential factors have been determined, by means of Equation (4), from the shift of peak temperature *T_n_* in the MS tests with different resonance frequencies, and their values are reported in Table 2. Seven different frequencies were used to get an Arrhenius plot of good quality.

In the 1st run a significant scattering of data was observed above ~230 °C; this effect is due to an irreversible transformation of the microstructure that is more evident in the 2nd run, where the blue symbols represent the difference between experimental data and fitting curve.

Moreover, the curves in the 1st and 2nd runs exhibit other relevant differences related to the intensities of the relaxation peaks: (i) in the 2nd MS test run the intensity of the peak *P*4 does not change; (ii) the peaks *P*2 and *P*3 are no longer present; and (iii) the intensity of *P*1 is strongly reduced. Such different behavior depends on the origin of each peak and provides information about some specific microstructural aspects.

**Figure 3:**
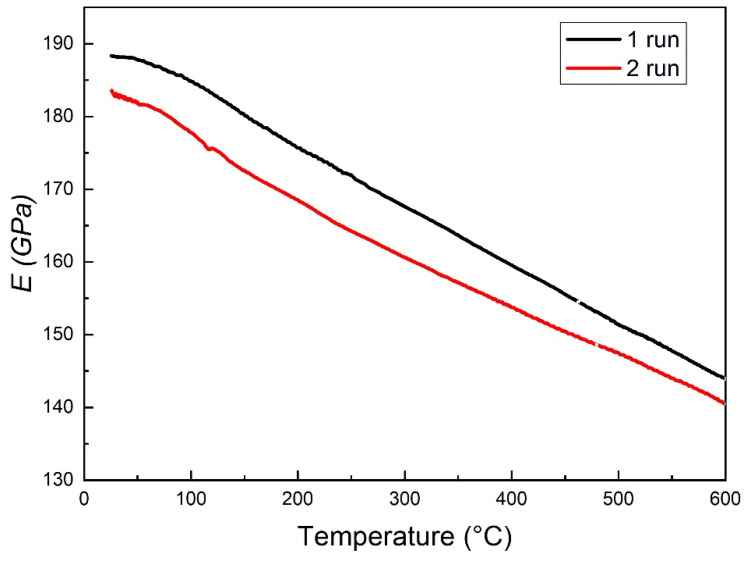
Trends of dynamic modulus *E* in the 1st and 2nd MS test runs.

The trends of dynamic modulus *E* in the 1st and 2nd MS test runs are displayed in Figure 3. They are similar in both the curves: *E* progressively decreases with temperature due to anharmonicity effects. However, after the first MS test run *E* is approximately 2% lower than its original value (188 GPa), confirming that microstructural changes occurred during the 1st run.

**Figure 4:**
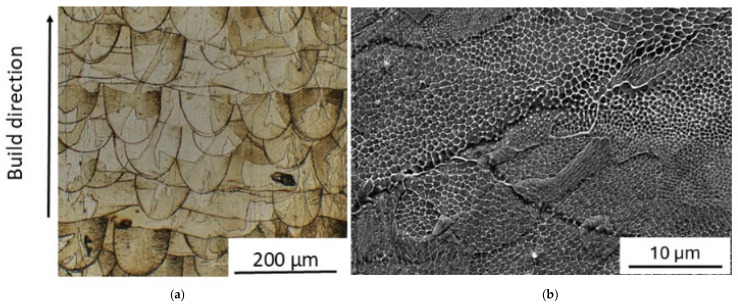
Microstructure of a sample in as-built condition. Typical pattern of melt pools and coarse columnar austenitic grains elongated in the build direction (**a**). At higher magnification a finer sub-structure of cells is observed (**b**).

The microstructure of as-printed material is shown in Figure 4. No pores, cracks, and unmelted powder particles are present in the as-built material. It exhibits the typical pattern of melt pools (width ≈ 90 µm and depth ≈ 110 µm) and coarse columnar austenitic grains (~150 μm), which are elongated in the build direction and not confined to a single deposited layer (Figure 4a). Some grains have a tortuous shape originating from the epitaxial growth through layers combined with rotations between each layer during the printing steps. As demonstrated by Pham et al. [49], this is due to side-branching growth. At higher magnification (Figure 4b), a finer sub-structure of cells with an average size of 0.4 μm is observed.

**Figure 5:**
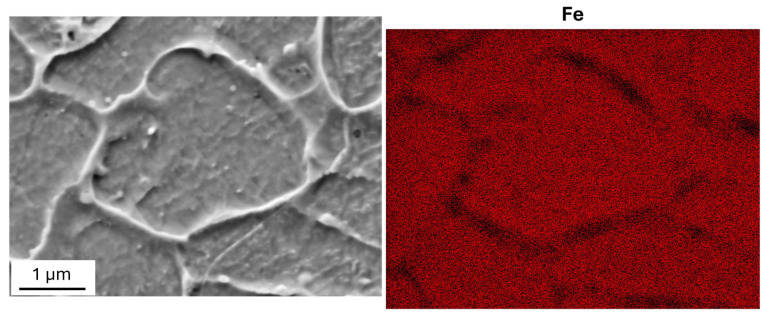

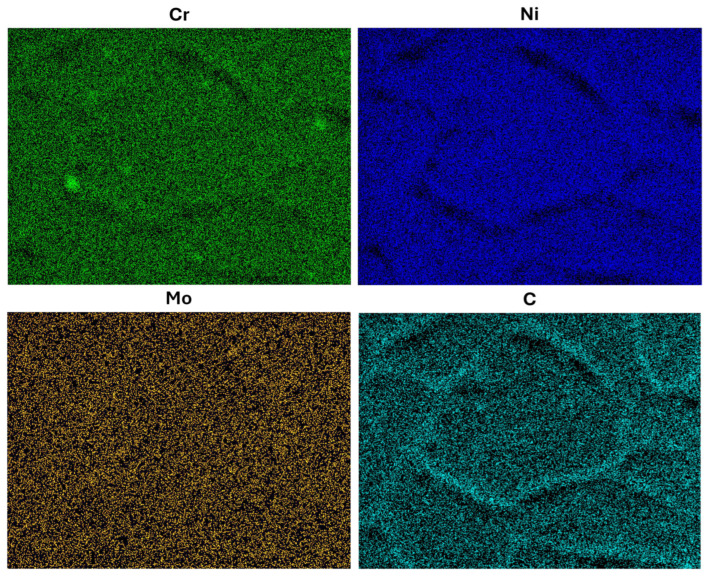
EDS maps of main alloying elements show that C is concentrated in the cell walls, which are impoverished of iron and nickel. The distribution of the other alloying elements is substantially homogeneous inside the grains.

As shown by the EDS maps in Figure 5, the cell walls exhibit a high concentration of carbon and are impoverished of iron and nickel, while the distribution of the other alloying elements is substantially homogeneous inside the grains. Of particular relevance is the homogeneous distribution of chromium, which could affect the corrosion behavior.

**Figure 6:**
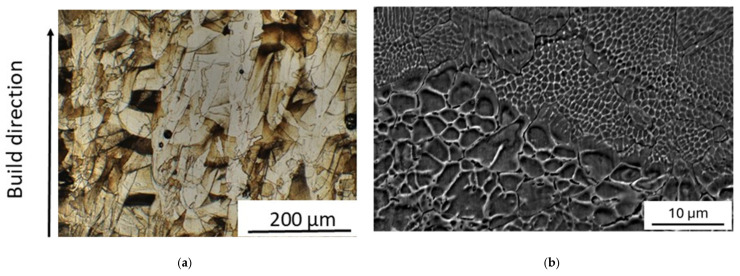
After MS tests the melt-pool pattern disappears and the elongated grains are substituted in large part by equiaxed ones (**a**). The average size of cells increases (**b**).

After MS tests, the melt-pool pattern disappears, and the elongated grains are substituted in large part by a population of equiaxed ones (Figure 6a). Although the average size of cells increases (average size of 0.8 μm), it is not homogeneous on the surface (Figure 6b), indicating the discontinuous evolution of the process.

**Figure 7:**
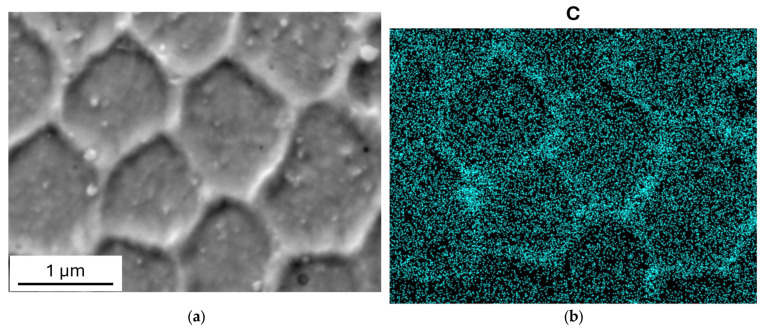
SEM image (**a**) and EDS map of carbon (**b**) post MS tests. The EDS map of carbon shows that it is still concentrated in the cell walls after MS tests.

As shown in Figure 7, carbon is still present in higher concentration in the cell walls after MS tests, while the distribution of other elements undergoes small changes.

**Figure 8:**
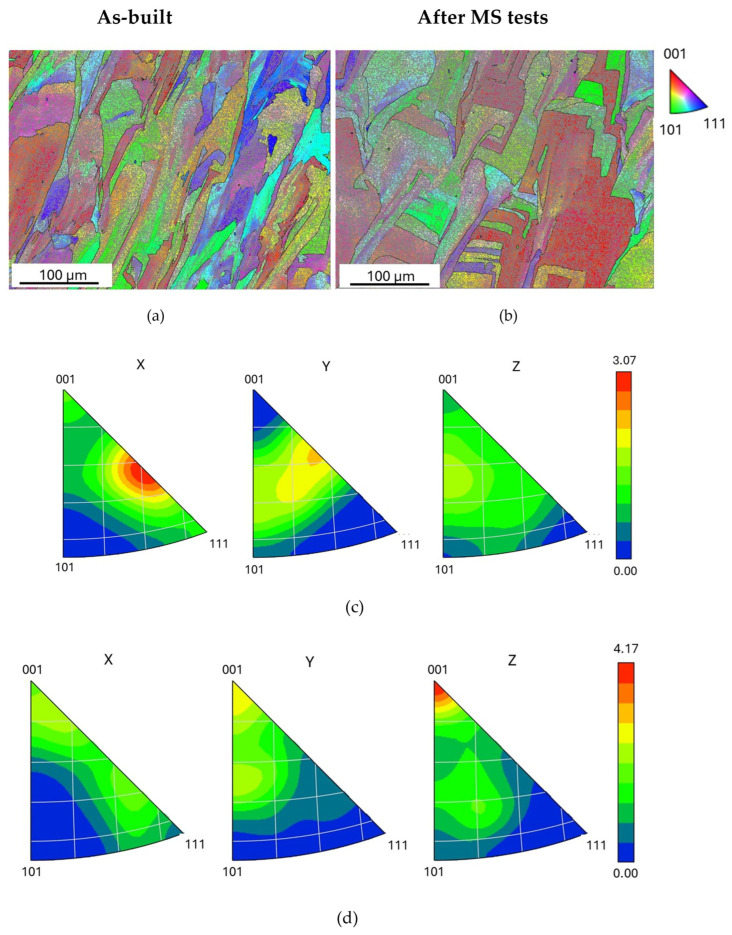
EBSD maps (**a**,**b**) and IPFs (**c**,**d**) of the as-built material (**a**,**c**) and after MS tests (**b**,**d**).

Figure 8 shows the maps and inverse polar figures (IPFs) obtained from EBSD measurements and analysis made on the material before and after MS tests. The EBSD maps in Figure 8a,b show grains with a color nuance in their interior corresponding to the fine cell structure. The dislocation walls between cells consist of low-angle grain boundaries (LAGBs, 2 < θ ≤ 15°). The distribution of grain-boundary misorientations in as-built steel evidences that there is a large prevalence (94.6%) of LAGBs in relation to high-angle grain boundaries (HAGBs, θ > 15°). The fraction of LAGBs does not change after MS test runs.

**Figure 9:**
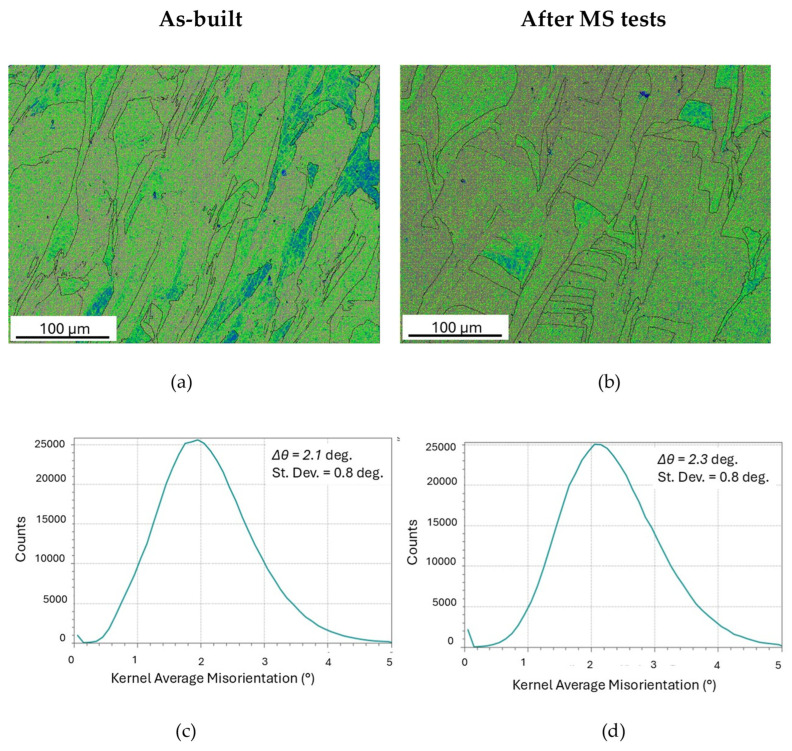
KAM maps (**a**,**b**) and average misorientation distributions (**c**,**d**) of the as-built material (**a**,**c**) and after MS tests (**b**,**d**).

KAM maps and average misorientation distributions before and after MS tests are displayed in Figure 9.

Both KAM maps (a,b) do not exhibit local stress concentrations. From the average values *Δθ*, expressed in radians, of KAM distributions in Figure 9c,d, the geometrically necessary dislocation density ζ_GND_ has been determined through Equation (5):

(5) $$\zeta_{GND}=\frac{k\Delta \theta }{b\Delta x}$$

where *b* = 0.2542 nm is the modulus of Burgers vector, *Δx* = 0.5 µm the distance over which the misorientation is measured and *k* a constant whose value depends on the type dislocations forming the boundary (*k* = 1 edge dislocations and *k* = 2 screw dislocations) [50,51]. By assuming that dislocations are of edge type, *k* = 1 was used in the present calculations. ζ_GND_ is 2.88 × 10^14^ m^−2^ in as-built material, a density similar to the ones reported in the literature [52,53], and 3.16 × 10^14^ m^−2^ after MS test runs. These values are very close and within the experimental error range (±1 × 10^14^ m^−2^); therefore, MS tests substantially very scarcely affect the dislocation density present in the as-printed steel.

**Figure 10:**
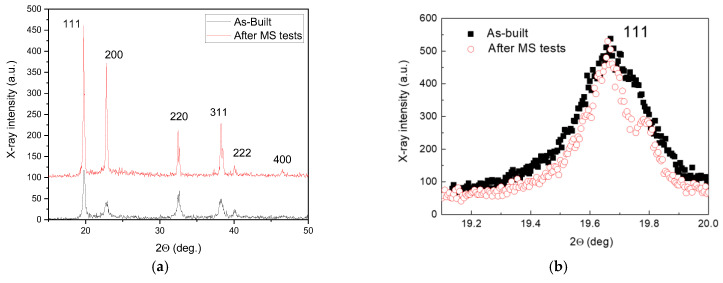
XRD patterns of AM 316 L in as-built condition and after MS tests (**a**). The comparison of the high precision {111} peak profiles shows a relevant narrowing after MS (**b**).

The evolution of the microstructure is also confirmed by XRD analysis, performed on the same sample before and after two MS test runs. The comparison between the XRD patterns (Figure 10a) shows a change in relative peak intensities, which depend on grain orientation. They are reported in Table 3, together and compared with the ones reported in the database JCPDS-ICDD describing the austenite with random oriented grains and considered as a reference. The peak intensities *I* are expressed as percentages of *I*_0_, the intensity of the strongest line on the pattern. In order to evaluate the texture, the parameter χ has been used (Equation (6)). It is defined as the ratio between the relative intensities of XRD peaks determined through experiments (*I*/*I*_0_)*_EXP_* and those of reference taken from the database JCPDS-ICDD, (*I*/*I*_0_)*_REF_*:


(6) $$\chi ={\left(\frac{I}{I_{0}}\right)}_{\mathrm{EXP}}/{\left(\frac{I}{I_{0}}\right)}_{\mathrm{REF}}$$

If the parameter χ of a generic set of planes {*hkl*} is greater than 1, there is a preferred grain orientation along the direction [*hkl*] and the texture becomes progressively stronger as the value of χ increases. As shown by the data in Table 3, the main texture component of as-built material is the [110] one with two secondary components, [111] and [311]. After the MS test runs, the cubic [100] component becomes prevalent while the [110], [311] and [111] result significantly weakened. In fact, this result confirms the texture modifications evidenced by EBSD.

Generally, the XRD peak positions in as-printed steel are shifted towards lower angles, indicating an expansion of the fcc lattice that is commonly attributed to the residual tensile stresses originating from the printing process [54]. An important aspect revealed by XRD is the narrowing of peak profiles; for example, the case of {111} reflection is shown in Figure 10. In Figure 10b, the central position has been normalized for the purpose of comparing the peak profiles.

## 4. Discussion

The present results show that the material undergoes significant changes during MS tests since the original microstructure is not stable due to the complex thermal history during printing operations, which consists of fast cooling of the melt and successive heating–cooling cycles of the solidified layers.

LM and SEM do not indicate the presence of ferrite, while in the XRD patterns very weak signals (intensity of the order of the background) were detected, corresponding with the {110} and {200} reflections; therefore, the amount of ferrite is very low and does not exceed 1–2%, namely the limit of XRD detectability. According to the Schaeffler diagram, the 316 L steel should be biphasic with about 5% of ferrite. Of course, this is what takes place in conditions of thermodynamic equilibrium, whereas the increase in cooling rate in L-PBF tends to shift the microstructure towards a single austenitic phase [55], but controversial experimental results can be found in the literature. Some interesting models have also been developed for predicting the microstructure of welds and also of AM printed austenitic steels [10]; however, foreseeing the final microstructure is a complex task due to the complex thermal history of the material.

As shown in Figure 4 and Figure 6, the original melt-pool pattern with elongated grains of the as-printed steel is replaced by a population of equiaxed grains, while the average size of cells increases. Such microstructural evolution is accompanied by a relevant texture change that leads the crystals to be mainly oriented along the [100] direction (see Table 3). The formation of new grains and the growth of cells necessarily involve the motion of both LAGBs and HAGBs, and this irreversible process gives rise to an increase in damping revealed in MS tests as an irregular signal, which is clearly visible in Figure 2a,b above ~230 °C. In the current literature (e.g., see ref. [52]), both the grain and cell structure are reported to be stable up to 500 °C, whereas the high sensitivity of MS highlights that the material starts to transform at lower temperature, and this information is very important for nuclear applications.

Another aspect of the microstructure evolution revealed by MS is related to the dislocation structures: KAM evidenced that the dislocation density does not change but MS shows a dynamic modulus decrease of about 2% after the first MS test run. According to the Granato-Lücke model [41], variations in dynamic modulus can be explained in terms of dislocation density ζ and average distance between dislocation pinning points *l* (Equation (7)):

(7) $$\frac{\Delta G}{G}\approx -\Gamma \zeta l^{2}$$

where *Γ* ≈ 1.67 is a constant. Although Equation (7) refers to the shear modulus *G*, the same effect also occurs when the dynamic modulus is *E* = 2*G*(1 + ν), with ν the Poisson’s ratio. Since the dislocation density is constant, the decrease in dynamic modulus can be ascribed to the increase in the average distance between dislocation pinning points. According to Equation (7), the decrease in dynamic modulus of about 2% corresponds to an increase of ≈15% in the average distance between dislocation pinning points. In fact, carbon atoms act as pinning points and, being mainly concentrated in cell walls (see EDS maps in Figure 5 and Figure 7), the cell growth leads to an increase in the distance *l*. With a view to finding further support for this explanation through direct observations of dislocation structures, transmission electron microscopy (TEM) experiments are underway.

As mentioned before, the damping vs. temperature curves (Figure 2) are the sum of an exponential background (*Q*^−1^*_back_*) plus four relaxation peaks whose characteristics are reported in Table 2.

The increasing background is generally due to thermally activated dislocation damping (e.g., see ref. [56]). For the purpose of understanding the origin of the peaks *P*1, *P*2, *P*3 and *P*4, their activation energies *H* and pre-exponential frequency factors *τ*_0_ were considered and are in the range expected for anelastic relaxations due to the motion of defects on atomic scale [41].

In an austenite lattice carbon atoms occupy the octahedral sites, which have cubic symmetry; thus, Snoek peaks are theoretically impossible. However, Snoek-like peaks in face-centered cubic lattices are reported in the literature because defect pairs give rise to deviations from the ideal interstitial defect symmetry. Defect pairs may consist of (1) two vacancies (V-V) [57,58]; (2) two interstitial atoms (I-I) [58,59]; (3) an interstitial atom and a vacancy (I-V) [60]; or (4) a substitutional and an interstitial atom (S-I) [61]. An exhaustive review of relaxation peaks originating from the re-orientation of various defect pairs under an external cyclic stress is presented in the book by Blanter et al. [42].

On the basis of the activation energies of peaks observed in the present experiments, they are not due to the re-orientation of C-C pairs because this process involves much higher *H* values (e.g., see refs. [58,62]). This view is confirmed by Mössbauer and in other studies about austenitic stainless steels under different conditions (deformed and annealed) that reveal that carbon atoms are randomly distributed and well separated in the matrix [63,64]. Moreover, the carbon content of 316 L is quite small (0.024 wt.%) and carbon atoms are mainly concentrated in cell walls; thus, a possible peak due to C-C pairs would be of negligible intensity.

Similar considerations apply to Cr-C pairs: the Finkelshtein–Rosin peak arising from the re-orientation of S-I pairs and usually exhibits an activation energy close to that of the interstitial diffusion and that for carbon diffusion in austenite *H* ≈ 30,000 cal/mol [65]); namely, almost twice that of the greatest one observed by us (peak *P*1, *H* = 15,300 cal/mol). However, the affinity of carbon for chromium, being stronger than for iron and nickel, is a key issue when explaining the origin of the observed anelastic phenomena. As shown by Oddershede et al. [66], through Extended X-ray Absorption Fine Structure (EXAFS) analysis, short range ordering of carbon and chromium atoms occurs in 316 L steel. The same phenomenon was extensively investigated in martensitic steels by some of the present authors [67,68], who found clear evidence of its impact on mechanical properties, in particular damping [69] and fracture behavior [70].

The interstitial sites, which could be occupied by carbon, have seven possible configurations corresponding to a number *i* of chromium atoms located at the corners of the octahedron, and varying from 0 to 6. The relative number *P*_i_ of octahedra with *i* chromium atoms is described by a binomial distribution:

(8) $$P_{i}=\frac{6!{\left(1-N\right)}^{6-i}N^{i}}{i!(6-i)!}$$

where *N* is the average chromium concentration (18.15 at. %). The fractions *P*_i_, calculated through Equation (8), are reported in Table 4, and it is evident that the distribution exhibits a maximum at *i* = 1 and results dramatically reduced for *i* ≥ 4.

In thermodynamic equilibrium, the concentrations *C_i_* of carbon atoms in octahedral interstices with *i* chromium neighbors depend on the total carbon concentration $C_{t}=\;\sum_{i=0}^{6}C_{i}=0.11\;at.\%$ and temperature. Details about the calculation of such distributions at the different temperatures can be found in the literature (e.g., see ref. [62]).

Octahedra with a high number of chromium atoms (5 and 6) are substantially saturated by carbon at room temperature but, nevertheless, as temperature increases, the partitioning of carbon tends to be more homogeneous; however, it remains mainly concentrated in the octahedra with high chromium. Since the fraction *P_i_* of *i-* type octahedra strongly decreases with *i* (Table 4), the concentrations *C_i_* with *i* ≤ 3 become prevalent at relatively low temperature (T < 470 °C).

**Figure 11:**
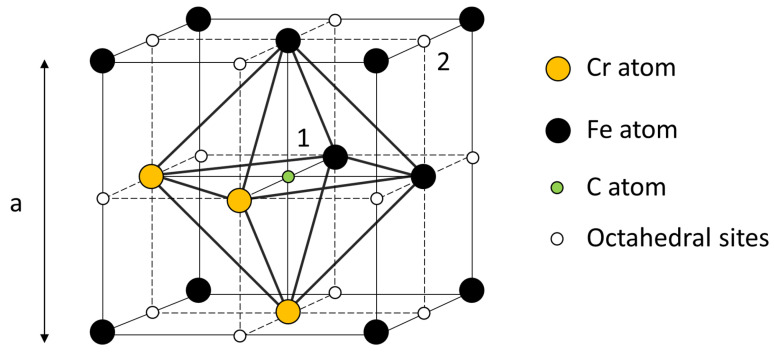
Unit cell of austenite, with lattice parameter *a*: three chromium atoms (yellow circles) are allocated at the corners of the octahedron around a carbon atom (green circle) in the fcc lattice. Detrapping consists of the jumping of carbon from position 1 (a trap) to position 2, which is surrounded by six iron atoms.

By investigating carbon diffusion in 316 L steel, Peng et al. [71] evidenced the trapping effect of chromium and considered a trapping–detrapping mechanism, determining a detrapping energy of 33 kJ/mol (7890 cal/mol). Figure 11 shows a carbon atom at the center of an octahedron with three chromium atoms at the corners (position 1). This position represents a trap, and detrapping means that the carbon atom jumps in position 2, which is not surrounded by chromium atoms. In fact, the value of detrapping energy corresponds to the activation energy of peak *P*4. Therefore, its origin can be ascribed to the alternate hopping of carbon atoms between positions 1 and 2. The intensity of the peak does not change in successive MS test runs because the number of carbon atoms in the traps generally remains constant.

A *Q*^−1^ peak with activation energy of the order 35 to 40 kJ/mole (8365–9560 cal/mol) was observed by Slane et al. [72] in austenitic Fe-34Ni alloys with different carbon content, ranging from 0.2 to 3.0 at. %, and ascribed to C-V pairs on the basis of first-principles density functional calculations. By investigating austenitic Fe-Mn-C-Al alloys, Young-Kook et al. [58] found two *Q*^−1^ peaks with activation energies *H* and relaxation times *τ*_0_*,* which were very close to those of *P*2 and *P*3, and which were, respectively, attributed to the re-orientation of V-V and C-V pairs. These pairs would generate strain fields with non-cubic symmetry and, consequently, their re-orientation under an external cyclic stress could originate relaxation peaks. Such an explanation is also consistent with the present results because *P*2 and *P*3 peaks substantially disappear in the second MS test run. As expected, the concentration of vacancies, in out-of-equilibrium conditions that are due to the fast cooling experienced by the material in printing operations, can re-equilibrate; namely, decreasing to the value corresponding to ambient temperature. Therefore, after the first MS test run, the strongly reduced concentration of vacancies involves a remarkable decrease in V-V and C-V pairs. Being the peak intensities proportional to the number of pairs participating in the re-orientation process, these peaks become of negligible intensity in the second MS run.

**Figure 12:**
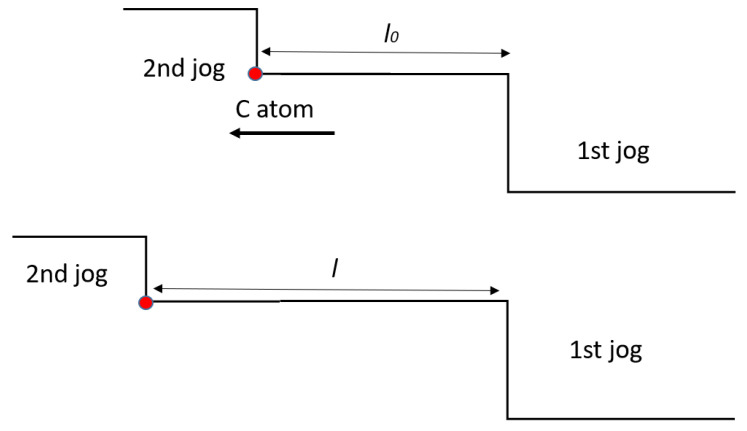
Schematic view of the mechanism that originates peak *P*1, involving the diffusion of C atoms along dislocation core. Owing to the movement of C atom, the length of the vibrating dislocation segment passes from *l*_0_ to *l*.

As compared to the other peaks, *P*1 has a greater activation energy (15,300 cal/mol). This value is consistent with the activation energy for pipe diffusion of carbon in austenitic steels. The density of dislocations in as-printed material is relatively high (2.88 × 10^14^ m^−2^), and it is commonly accepted that dislocations originate from thermal strains in the expansion/shrinkage cycles experienced by the material after solidification. Moreover, in additive manufacturing (AM) processes, the shrinkage of the melt pools during solidification is constrained by the surrounding previously deposited material. As a result, residual stresses may develop and locally exceed the yield strength, leading to plastic deformation that further increases the dislocation density. As assessed by many investigators (e.g., see refs. [33,35,52]), dislocations are mainly organized in the cell boundaries, whereas their density in the cell interiors is quite lower. Taking into account activation energy and specific dislocation structure, it is reasonable that the peak *P*1 could be due to the vibration of dislocation segments anchored at jogs, which represent mobile pinning points whose movement is assisted by carbon pipe diffusion at the dislocation core. A schematic view of the mechanism is shown in Figure 12: the diffusion of the C atom along the dislocation line determines the length *l* of the dislocation segment between the 1st and 2nd jogs and the vibration through which such segment contributes to damping.

At low frequencies, the damping contribution due to a single dislocation segment can be expressed by Equation (9) [41]:

(9) $$Q^{-1}=\frac{\zeta Bl^{4}}{36\;Gb^{2}}\omega$$

where *B* is the dislocation damping parameter. It is evident from Equation (9) that small variations in *l* lead to relevant changes of *Q*^−1^. The partial disaggregation of dislocation walls, with consequent cell growth occurring in the first MS test run, on the one hand increases the average value of *l* and, on the other hand, reduces the number of jogs. The second aspect seems to be prevalent because the peak intensity decreases. The idea that the movement of jogs assisted by pipe diffusion may give rise to a relaxation peak was originally presented in a paper by Choi and Nix [73], who developed a theoretical model to explain the anelastic behavior of thin copper films. More recently, through MS, one of the present authors detected the same phenomenon in Al foils and showed, by means of TEM observations, the presence of jogs [74]. Anyway, the given explanation of peak *P*1 still remains a hypothesis that needs to be confirmed by TEM observations, which are underway.

## 5. Conclusions

This work was carried out to assess the microstructural stability of 316 L steel produced by L-PBF, and it demonstrated that it is highly unstable. Moreover, MS tests evidenced some new aspects not yet reported in the current literature. The main results can be summarized as follows.

(i) The typical melt-pool pattern, with grains elongated in the build direction and not confined to a single deposited layer, is progressively substituted by a population of equiaxed grains. The average size of the cells forming a finer sub-structure inside the grains increases. Grain and cell evolution is accompanied by a texture change.
(ii) Although the current literature reports that grain and cell structure is stable up to 500 °C, MS results indicate that the aforesaid irreversible phenomena begin to take place at a lower temperature (~230 °C).
(iii) The original microstructure involves a vacancy concentration out of the thermodynamic equilibrium and the excess of vacancies form V-V and C-V pairs. A single MS test run is sufficient to re-equilibrate vacancy concentration and suppress related anelastic phenomena.
(iv) The dislocation density present in the printed steel is scarcely affected and the decrease in dynamic modulus, observed after the first MS run, has been ascribed to the increase in the mean distance between dislocation pinning points. Such distance changes occur by means of carbon diffusion along dislocation cores. The proposed mechanism needs to be confirmed by TEM observations, which are underway.

Since the identified ~230 °C stability threshold is lower than the operating temperatures of critical EU DEMO components like the Vacuum Vessel and cooling manifolds (e.g., water at 295 °C), present results suggest that the microstructure of the printed material should be stabilized through suitable heat treatments before its application in structural components of nuclear reactors.

## Figures and Tables

**Table 1:** Chemical composition of the powder used to manufacture the samples (wt. %).

C	Cr	Mo	N	Mn	Si	Ni	P	S	Fe
0.024	16.87	2.06	0.083	1.35	0.40	10.05	0.031	0.029	to balance

**Table 2:** Activation energies *H_n_*, pre-exponential frequency factors *τ*_0_ and relaxation strength *Δ_n_* of the Debye peaks (P1, P2, P3 and P4) used to fit the curves in Figure 2a,b obtained in the 1st and 2nd MS test run, respectively.

Peak	*P*1	*P*2	*P*3	*P*4
*H_n_* (cal/mol)	15,300	13,512	11,127	8345
*τ*_0_ (s)	10^−12 ± 2.1^	10^−7 ± 1.5^	10^−6 ± 1.4^	10^−14 ± 1.9^
*Δ_n_* (1st run)	1.8 × 10^−3^	1.26 × 10^−3^	0.6 × 10^−3^	2.4 × 10^−3^
*Δ_n_* (2nd run)	0.2 × 10^−3^	-	-	2.4 × 10^−3^

**Table 3:** Relative intensities of the strongest XRD peaks of 316 L steel in as-built condition and after the MS test runs. The peak intensities *I* are expressed as percentages of *I*_0_, the intensity of the strongest line on the pattern. The parameter χ, defined in Equation (6), describes the texture.

	Peaks	{111}	{200}	{220}	{311}	{222}	{400}
As-built	*I*/*I*_0_	100	35	58	42	21	-
	χ	1	0.78	2.23	1.40	1.75	-
After MS tests	*I*/*I*_0_	100	75	31	36	8	6
	χ	1	1.67	1.19	1.20	0.67	2.0
JCPDS-ICCD	*I*/*I*_0_	100	45	26	30	12	3

**Table 4:** Fractions of octahedra with a number of chromium atoms varying from 0 to 6, as calculated by Equation (8).

Octahedra Type	*P*_0_	*P*_1_	*P*_2_	*P*_3_	*P*_4_	*P*_5_	*P*_6_
Fraction	0.30	0.40	0.2218	6.56 × 10^−2^	1.09 × 10^−2^	9.67 × 10^−4^	3.57 × 10^−5^

## Data Availability

The original contributions presented in this study are included in the article. Further inquiries can be directed to the corresponding author.

## Abbreviations

Symbol	Nomenclature
*E*	Dynamic modulus
*ρ*	Material density
*L*	Length of the sample
*h*	Thickness of the sample
*f*	Resonance frequency
*m*	Constant equal to 1.875
*Q* ^−1^	Damping parameter
*A_n_*	Amplitude of n-th oscillation
*A_n_*_+_ *_p_*	Amplitude of n + p-th oscillation
*Θ*	Diffraction angle
*Δ_n_*	Relaxation strength of the n-th peak
*H_n_*	Activation energy of the n-th peak
*R*	Gas constant
*T*	Temperature
*T_n_*	Temperature of the central position of n-th peak
*Q* ^−1^ *_back_*	Exponential background
*τ*	Relaxation time
*τ*_0_	Pre-exponential factor of the relaxation time
*ζ_GND_*	Geometrically necessary dislocation density
*k*	Constant whose value depends on the type of dislocations forming the boundary (k = 1 edge dislocations, k = 2 screw dislocations)
*b*	Modulus of Burgers vector, b = 0.2542 nm
*θ*	Misorientation angle
*Δx*	Distance over which the misorientation is measured
χ	Texture parameter
(*I*/*I*_0_)*_EXP_*	Experimental relative intensity
(*I*/*I*_0_)*_REF_*	Reference relative intensity
*Γ*	Constant equal to 1.67
*ζ*	Dislocation density
*P_i_*	The relative number of octahedra with i chromium atoms
*i*	Number of chromium atoms in one octahedron
*N*	Average chromium concentration
*C_i_*	Carbon atoms in octahedral interstices with i chromium neighbors
*C_t_*	Total carbon atoms
*B*	Dislocation damping parameter
*l*	Average distance between dislocation pinning points
*G*	Shear modulus
